# Expression and Impact of Adenosine A_3_ Receptors on Calcium Homeostasis in Human Right Atrium

**DOI:** 10.3390/ijms24054404

**Published:** 2023-02-23

**Authors:** Carmen Tarifa, Verónica Jiménez-Sábado, Rafael Franco, José Montiel, José Guerra, Francisco Ciruela, Leif Hove-Madsen

**Affiliations:** 1 Biomedical Research Institute of Barcelona, IIBB-CSIC, 08036 Barcelona, Spain; 2 Institut d’Investigació Biomèdica Sant Pau (IIB Sant Pau), 08025 Barcelona, Spain; 3 Cardiology Department, Hospital de la Santa Creu i Sant Pau, 08025 Barcelona, Spain; 4 Centro de Investigación Biomédica en Red de Enfermedades Cardiovasculares (CIBERCV), Instituto de Salud Carlos III, 28029 Madrid, Spain; 5 Departament de Bioquímica i Biomedicina Molecular, Facultat de Biología, Universitat de Barcelona, 08028 Barcelona, Spain; 6 Cardiac Surgery Department, Hospital de la Santa Creu i Sant Pau, 08025 Barcelona, Spain; 7 Pharmacology Unit, Department of Pathology and Experimental Therapeutics, Faculty of Medicine and Health Sciences, Institute of Neurosciences, University of Barcelona, 08907 L’Hospitalet de Llobregat, Spain; 8 Neuropharmacology & Pain Group, Neuroscience Program, Institut d’Investigació Biomèdica de Bellvitge, IDIBELL, 08907 L’Hospitalet de Llobregat, Spain

**Keywords:** human atrial myocyte, adenosine A_3_ receptor, adenosine A_2A_ receptor, sarcoplasmic reticulum, calcium spark, transient inward current, L-type calcium current, electrophysiology

## Abstract

Increased adenosine A_2A_ receptor (A_2A_R) expression and activation underlies a higher incidence of spontaneous calcium release in atrial fibrillation (AF). Adenosine A_3_ receptors (A_3_R) could counteract excessive A_2A_R activation, but their functional role in the atrium remains elusive, and we therefore aimed to address the impact of A_3_Rs on intracellular calcium homeostasis. For this purpose, we analyzed right atrial samples or myocytes from 53 patients without AF, using quantitative PCR, patch-clamp technique, immunofluorescent labeling or confocal calcium imaging. A_3_R mRNA accounted for 9% and A_2A_R mRNA for 32%. At baseline, A_3_R inhibition increased the transient inward current (I_TI_) frequency from 0.28 to 0.81 events/min (*p* < 0.05). Simultaneous stimulation of A_2A_Rs and A_3_Rs increased the calcium spark frequency seven-fold (*p* < 0.001) and the I_TI_ frequency from 0.14 to 0.64 events/min (*p* < 0.05). Subsequent A_3_R inhibition caused a strong additional increase in the I_TI_ frequency (to 2.04 events/min; *p* < 0.01) and increased phosphorylation at s2808 1.7-fold (*p* < 0.001). These pharmacological treatments had no significant effects on L-type calcium current density or sarcoplasmic reticulum calcium load. In conclusion, A_3_Rs are expressed and blunt spontaneous calcium release at baseline and upon A_2A_R-stimulation in human atrial myocytes, pointing to A_3_R activation as a means to attenuate physiological and pathological elevations of spontaneous calcium release events.

## 1. Introduction

Cyclic AMP (cAMP) signaling plays a crucial role in modulating calcium regulatory proteins involved in cardiac excitation-contraction coupling, such as L-type calcium channels, the sarcoplasmic reticulum (SR) calcium channel, also named the ryanodine receptor (RyR2), and phospholamban that regulates the activity of the SR calcium pump [1,2]. Physiological and pathological modulation of cAMP signaling, in turn, involves a large number of G protein-coupled receptors (i.e., GPCRs) and phosphodiesterases [3,4]. Within GPCRs, adenosine receptors play a key role in the regulation of myocardial function and rhythm [5]. In this context, the Gi-protein coupled adenosine A_1_ receptor (A_1_R) is expected to reduce cAMP production and attenuate the sympathetic tone, and the A_1_R is a pharmacological target for the regulation of supraventricular arrhythmias [6]. However, excessive A_1_R activation can also accelerate atrial fibrillation (AF) [7] or favor its induction by shortening the refractory period via activation of the G-protein coupled inwardly rectifying potassium channel [8]. Furthermore, both the A_1_R and the Gi-protein coupled adenosine A_3_ receptor (A_3_R) has been attributed important roles in ischemic preconditioning and cardio protection [9,10,11]. Moreover, the adenosine A_2A_ receptor (A_2A_R) and A_2B_ receptor (A_2B_R) are Gs-protein coupled receptors that are expected to stimulate cAMP synthesis and favor cAMP-dependent phosphorylation of key calcium regulatory proteins. Indeed, the A_2A_R displays an overlapping distribution with the RyR2 and has previously been shown to selectively modulate spontaneous calcium release from the SR [12]. Moreover, A_2A_R expression is upregulated in patients with AF and prevention of A_2A_R activation normalizes spontaneous calcium release in patients with AF to levels observed in patients without AF [13], and diminishes the induction of arrhythmic responses in electrically paced myocytes from patients with AF [14]. Because the A_3_R is expected to inhibit adenylate cyclase, activation of this receptor would be expected to dampen spontaneous A_2A_R-induced calcium release and contribute to maintaining a low incidence of spontaneous calcium release at baseline. Currently, the functional role of A_3_Rs in atrial myocytes remains elusive, and there are notable differences in A_3_R expression or binding of agonists to A_3_Rs in atria from humans and small rodents [15]. However, since there are species-dependent differences in the expression of G-protein coupled receptors and their binding constants for A_3_R agonists [15,16], this study aims to determine the expression of A_3_Rs in human right atrial samples and the functional impact on intracellular calcium homeostasis in human right atrial myocytes.

## 2. Results

To determine the functional impact of A_3_Rs in the human atrium, we analyzed the expression and functional electrophysiological impact of A_3_Rs in myocytes from 53 patients without a previous history of AF. Table 1 summarizes the clinical features of the study population.

### 2.1. Adenosine A_3_R Expression

**Figure 1:**
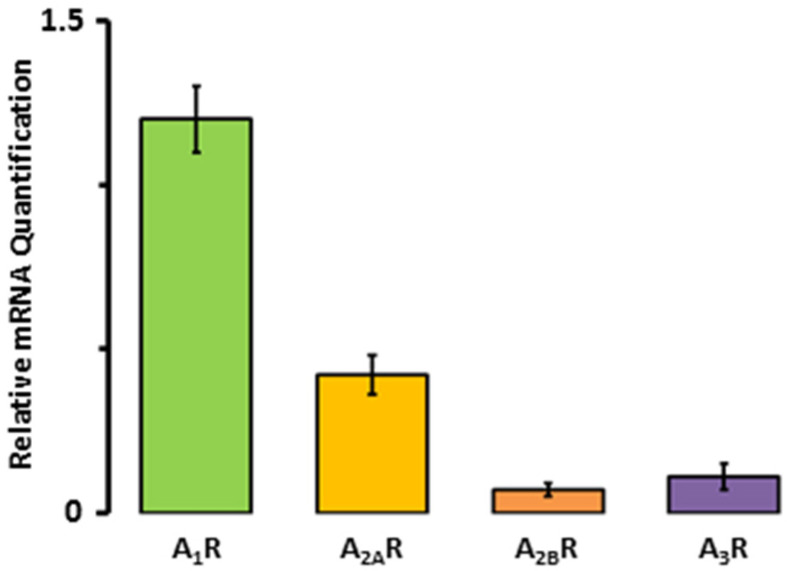
Adenosine receptor mRNA expression in human right atrial tissue samples from seven patients.

First, we aimed to determine the expression levels of A_3_R mRNA in comparison to the other adenosine receptors. The results shown in Figure 1 indicate that A_1_R mRNA is the most abundant, followed by the A_2A_R and the A_3_R. Specifically, the expression of A_2A_R mRNA constituted the 35 ± 5% of the A_1_Rs, while A_3_R accounted for only 9.2 ± 3.3%.

### 2.2. Impact of Adenosine A_3_Rs on Calcium Homeostasis at Baseline

**Figure 2:**
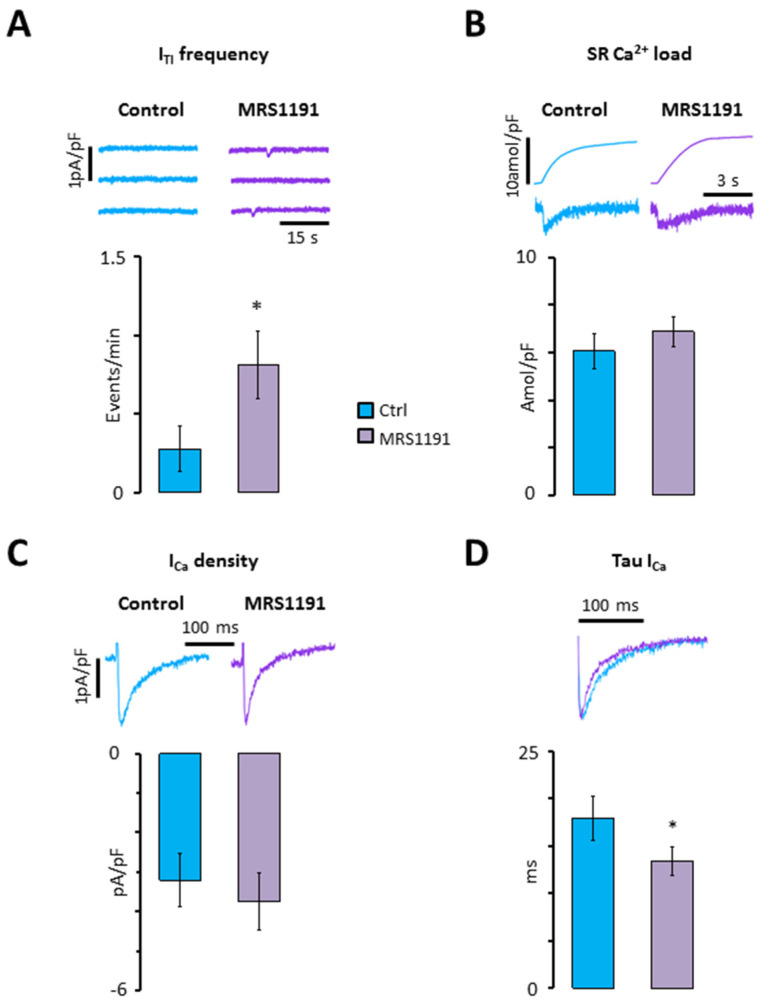
Impact of A_3_R inhibition on calcium homeostasis at baseline. (**A**) I_TI_ frequency; (**B**) SR calcium load; (**C**) I_Ca_ density; (**D**) tau of I_Ca_. Data were recorded in nine myocytes from eight patients before (blue) and after perfusion of myocytes with MRS1191 (purple). Significant differences are indicated with * *p* < 0.05 (paired *t*-test).

However, since GPCRs can be located in macromolecular clusters where they exert a strong regulation of specific molecular functions [17], we first determined how selective A_3_R inhibition affected calcium homeostasis at baseline. As shown in Figure 2A,B, the selective A_3_R antagonist MRS1191 significantly increased the incidence of I_TI_ (Figure 2A), without affecting the caffeine releasable SR calcium load (Figure 2B). Furthermore, MRS1191 did not have a significant effect on the I_Ca_ amplitude (Figure 2C), but significantly increased the I_Ca_ inactivation (Figure 2D).

### 2.3. Impact of Crosstalk between A_3_R and A_2A_R on Spontaneous Calcium Release

**Figure 3:**
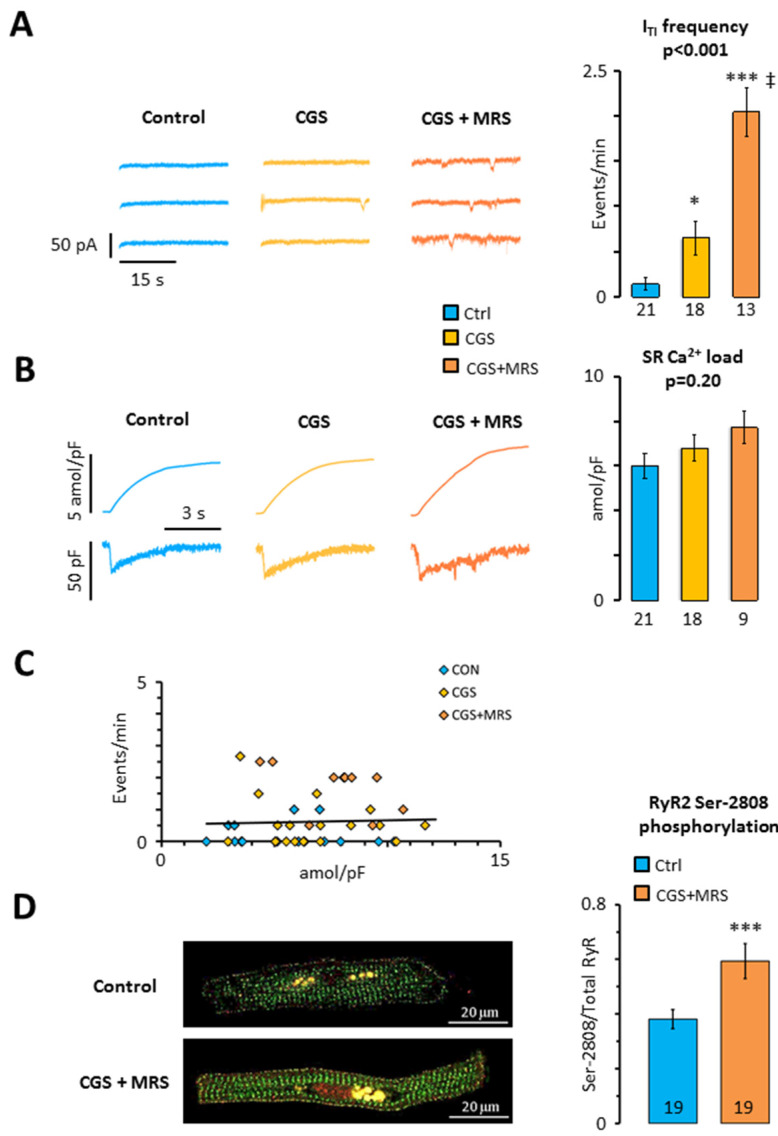
Impact of crosstalk between A_3_R and A_2A_R on SR calcium homeostasis. (**A**) I_TI_ frequency; (**B**) SR calcium load; (**C**) relationship between I_TI_ frequency and SR calcium load. Data were recorded in 21 myocytes from 19 patients before (blue) and after perfusion of myocytes with CGS21680 (yellow) and CGS21680 and MRS1191 (orange); (**D**) RyR2 s2808 phosphorylation in 106 myocytes from 19 patients. Significant differences between the control and treatments are indicated with * *p* < 0.05; *** *p* < 0.001, ‡ *p* < 0.01 for CGS21680 + MRS1191 vs. CGS21680. Data in (**A**,**B**) were analyzed using ANOVA with Welch’s correction (*p*-values are given above the bars) and Games–Howell post-test. Unpaired *t*-test was used in panel (**D**).

Since we have previously shown that A_2A_R activation contributes to a higher incidence of I_TI_ in human atrial myocytes [12] and A_3_R activation would counteract this, we tested whether there is a crosstalk between A_3_Rs and A_2A_Rs in human atrial myocytes. For this purpose, we first exposed human atrial myocytes to 200 nM CGS21680 to simultaneously stimulate A_2A_Rs and A_3_Rs. As shown in Figure 3A, this significantly increased the I_TI_ frequency four-fold. Interestingly, subsequent exposure to MRS1191 induced an additional 3-fold increase in the I_TI_ frequency (*p* < 0.001), suggesting that the A_3_R blunts the effect of A_2A_R activation. Analysis of the caffeine-releasable SR calcium load revealed that the treatment with CGS21680 or CGS21680 + MRS1191 did not affect the SR calcium load significantly (Figure 3B), suggesting that the increased incidence of I_TI_ is not caused by a higher SR calcium load. Accordingly, there was no significant correlation between SR calcium load and I_TI_ frequency (*p* = 0.789; Figure 3C). Immunofluorescent labeling of the RyR2 phosphorylated at s2808 revealed that phosphorylation was significantly higher in myocytes incubated with CGS21680 + MRS1191 than in control myocytes from the same patient (Figure 3D), suggesting that s2808 phosphorylation could contribute to the higher incidence of I_TI_ observed after exposure to CGS21680 + MRS1191.

**Figure 4:**
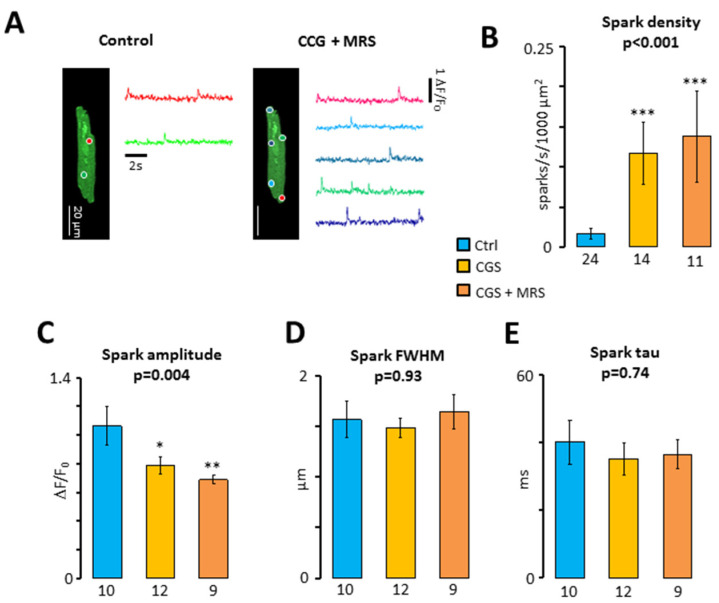
Impact of the crosstalk between A_3_R and A_2A_R on calcium sparks. (**A**) Calcium spark recordings (colored traces) from their respective spark sites (colored circles) in a patient before and after exposure to 200 nM CGS21680 + 1 μM MRS1191; (**B**) spark density (sparks/s/1000 μm^2^). Sparks were recorded in 10 patients before (blue) and after perfusion of myocytes with CGS21680 (yellow) and CGS21680 and MRS1191 (orange). Number of cells are given below bars; (**C**) spark amplitude (dF/F_0_); (**D**) spark full width at half maximum (FWHM, μm); (**E**) tau for spark decay (ms). Significant differences between the control and treatments are indicated with* *p* < 0.05; ** *p* < 0.01; *** *p* < 0.001. Data were analyzed using Kruskal–Wallis (*p*-values are given above Bars) and Dunn’s post-test in (**B**) and ANOVA with Welch’s correction in panel (**C**–**E**). Number of cells with sparks are given below the bars in panel (**C**–**E**).

To determine whether A_2A_R activation increases the I_TI_ frequency by increasing the propensity of the RyR2 to open spontaneously, we analyzed the incidence of calcium sparks resulting from the opening of individual RyR2 clusters [18]. Figure 4A,B shows that both CGS21680 and CGS21680 + MRS1191 induced a dramatic increase in the calcium spark density. This concurred with a significant reduction of the calcium spark amplitude, which was most pronounced in the presence of CGS21680 + MRS1191 (Figure 4C). On the contrary, the treatments had no significant impact on the width (Figure 4D) or the decay of the calcium sparks (Figure 4E).

Table 2 summarizes the impact of the two treatments on all calcium spark features analyzed.

### 2.4. Impact of Crosstalk between A_3_R and A_2A_R on L-type Calcium Current

**Figure 5:**
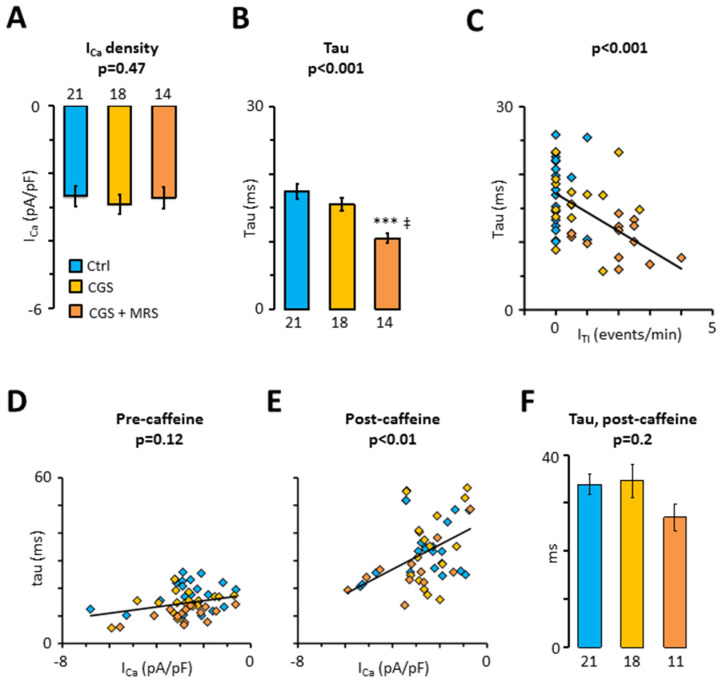
Impact of the crosstalk between A_3_R and A_2A_R on L-type calcium current. (**A**) I_Ca_ density; (**B**) fast time constant, tau, for I_Ca_ decay; (**C**) correlation between tau and I_TI_ frequency (from Figure 3A); (**D**,**E**) correlation between tau and I_Ca_ density before and after transient exposure to 10 mM caffeine. Currents were recorded in 19 patients before (blue) and after perfusion of myocytes with CGS21680 (yellow) and CGS21680 + MRS1191 (orange). Statistical significance was evaluated using Pearson’s product-moment correlation; (**F**) Time constant, tau, for the decay of the first I_Ca_ after transient exposure to caffeine. Significant differences between control and treatments are indicated with; *** *p* < 0.001, ‡ *p* < 0.01 for CGS21680 + MRS1191 vs. CGS21680. Data were analyzed using ANOVA (*p*-values are given above the bars) and Tukey’s post-test in (**A**,**B**,**F**). Number of cells are given below bars.

Finally, the treatment with CSG21680 and CGS21680 + MRS1191 was used to assess the impact of A_2A_Rs and A_3_Rs on I_Ca_. Figure 5A shows that neither A_2A_R nor A_3_R activation had any impact on I_Ca_ amplitude. However, concurrent activation of A_2A_Rs and inhibition of A_3_Rs with CGS21680 + MRS1191 significantly increased time-dependent inactivation of I_Ca_ (Figure 5B). Moreover, Figure 5C shows that the time constant for fast I_Ca_ inactivation (tau) was inversely correlated with the I_TI_ frequency recorded in the same cell (*p* < 0.001). In contrast, Figure 5D showed only a weak correlation between tau and I_Ca_ density, suggesting that I_Ca_ inactivation by calcium influx through the proper L-type calcium channel is modest. However, Figure 5E shows that a brief transient exposure to caffeine, to eliminate SR calcium release-induced I_Ca_ inactivation, unmasks a steeper correlation between tau and the I_Ca_ density (*p* < 0.01). Even so, Figure 5F shows that the tau for the I_Ca_ inactivation elicited after caffeine exposure is not modified by the treatments with CGS21680 and MRS1191, suggesting that A_2A_Rs and A_3_Rs have a minor impact on I_Ca_ amplitude or inactivation.

## 3. Discussion

### 3.1. Main Findings

While the A_3_R has been attributed an important role in preconditioning and cardio protection [10,19], little is known about its functional role in human atrial myocytes. Here, we analyzed the impact of the A_3_R on intracellular calcium homeostasis and report that even though A_3_R mRNA expression is modest compared to the expression of the A_1_R and the A_2A_R, endogenous activation of the A_3_Rs at baseline blunts the incidence of the spontaneous calcium release-induced I_TI_. Furthermore, crosstalk between A_3_R and A_2A_R upon activation of both receptors reduces the incidence of both calcium sparks and I_TI_, demonstrating that A_3_R activation diminishes spontaneous, A_2A_R-mediated, calcium release in human atrial myocytes. The findings also suggest that A_3_R activation could be a means of attenuating arrhythmogenic calcium release events induced by pathological elevations of the adenosine level.

### 3.2. Impact of the A_3_R on Calcium Homeostasis at Baseline

Previous electrophysiological studies in human atrial myocytes have reported a cAMP-tonus at baseline [20], which is regulated by phosphodiesterases and modulates I_Ca_ amplitude [21] as well as the incidence of spontaneous calcium release [22]. Consistent with this, we have also shown that the ruptured whole-cell patch configuration dialyses adenosine out of the cell, leading to a reduction of the I_TI_ frequency, presumably because the endogenous adenosine level is sufficient to induce spontaneous, A_2A_R-mediated, calcium release at baseline [13]. In accordance with these findings, we here observe that selective inhibition of the A_3_R with MRS1191 increases the basal I_TI_ frequency, suggesting that endogenous adenosine not only activates A_2A_Rs, but also A_3_Rs at baseline, and that the latter attenuates A_2A_R-mediated activation of adenylate cyclase. Interestingly, we do not observe any significant effect of A_3_R inhibition on I_Ca_ density or SR calcium loading, pointing to compartmentalization of A_3_R-mediated signaling. This finding is similar to previous observations on the impact of pharmacological manipulation of Gs-protein coupled receptors in human atrial myocytes where acute pharmacological manipulation of A_2A_R or treatment of patients with β-adrenergic receptor blockers had no impact on I_Ca_ density or SR calcium load [12,13,23].

### 3.3. Impact of Crosstalk between A_3_Rs and A_2A_Rs on Calcium Homeostasis

Since AF has previously been associated with increased A_2A_R expression and activation that promotes spontaneous calcium release [13], concurrent activation of the A_3_R would be expected to dampen A_2A_R-mediated stimulation of calcium release. The present findings demonstrate that when A_2A_Rs and A_3_Rs are activated simultaneously, A_3_R activation does indeed attenuate significantly the A_2A_R-mediated increase in the incidence of both calcium sparks and I_TI_. In support of this finding, both A_3_R and A_2A_R bind adenosine with an affinity of approximately 300 nM [24]. Similar to observations at baseline, A_3_R inhibition did not modify the I_Ca_ density when the A_2A_Rs and A_3_Rs were stimulated simultaneously with CGS21680. However, A_3_R inhibition did speed up I_Ca_ inactivation and this was correlated with the I_TI_ frequency but not with the I_Ca_ amplitude recorded in the same cell. This, combined with the higher incidence of calcium sparks and increased RyR2 phosphorylation at s2808 observed upon concurrent activation of A_2A_R and A_3_R suggests that the A_3_R selectively targets cAMP-dependent phosphorylation of the RyR2 and that this not only leads to a higher I_TI_ frequency, but also leads to a faster calcium-release induced I_Ca_ inactivation. Interestingly, the tau for I_Ca_ inactivation was inversely proportional to the I_Ca_ amplitude when cells had previously been exposed to caffeine to clear the SR calcium content and prevent calcium-release induced inactivation. However, even under these conditions, CGS21680 or CGS21680 + MRS1191 did not affect the I_Ca_ amplitude significantly, confirming that A_2A_R and A_3_R-dependent signaling targets the RyR2 but not the L-type calcium channel.

### 3.4. Study Limitations

In the present study, we have focused on the impact of A_3_Rs on intracellular calcium homeostasis. However, being a Gi-protein coupled receptor, it is conceivable that the A_3_Rs could also modulate the activity of other ion channels that are regulated by Gi-protein coupled receptors [8,9] or influence the activity of other Gs-protein coupled receptors [25]. This, in turn, would potentially influence the net impact of A_3_R activity on the amplitude and frequency of calcium release-induced afterdepolarizations. Similarly, the relative impact of A_3_Rs on A_2A_R-mediated signaling will depend on the spatial distribution of the A_1_R, the A_2A_R, and the A_3_R with respect to target proteins, such as the RyR2, L-type calcium channels, phospholamban, etc. While this issue has been addressed in non-myocardial preparations [25,26,27], such information is currently limited for atrial myocytes. In this regard, we did show that a gradual elevation of intracellular adenosine levels to pathological levels strongly increases the incidence of calcium waves and I_TI_ and that this could be reversed by selective A_2A_R inhibition [13], suggesting that A_2A_R activation plays a prominent role in pathological elevations of the adenosine level. Moreover, this study uses human atrial myocytes that are well suited for translational studies of receptor-mediated modulation of electrophysiological function. However, we cannot rule out that our findings could potentially be affected or present variability due to variations in concurrent disease, risk factors, or pharmacological treatments among the study population. In this context, age has been shown to affect I_Ca_ density [28] and sex has been shown to have differential effects on I_Ca_ density and I_TI_ frequency [29]. However, because this study does not compare different groups of patients, this issue is primarily expected to increase variability between measurements, rather than the effect of pharmacological treatments. Similarly, the present study was conducted in right atrial myocytes, and we cannot rule out that some of the findings may be specific to the right atrium.

### 3.5. Clinical Implications and Conclusion

We have previously reported that the expression of A_2A_Rs is upregulated in AF, and underlies a higher incidence of calcium-release induced I_TI_ and afterdepolarizations in atrial myocytes from patients with AF [13]. The relevance of these findings is further underscored by higher plasmatic adenosine levels and lower adenosine deaminase activity in patients with AF [30]. In this context, the present findings, showing that A_3_R activation regulates the impact of A_2A_R activation on RyR2 phosphorylation and spontaneous calcium release, suggest that selective activation of adenosine A_3_Rs might be suitable to dampen pathological elevations of the incidence of spontaneous calcium release-induced electrical activity. In particular, cardioprotective approaches targeting the A_3_R during ischemia, where adenosine levels are known to surge [31], could be a point of departure to explore the potential of A_3_Ra as a novel target to prevent induction of atrial ectopic atrial activity.

## 4. Materials and Methods

### 4.1. Myocyte Isolation

Atrial myocytes were isolated from tissue fragments collected from 53 patients, without a previous history of AF, undergoing cardiac surgery at Hospital de la Santa Creu i Sant Pau in Barcelona. Clinical and echocardiographic data of these patients are summarized in Table 1. Myocytes were isolated from right atrial samples, as previously described [28]. Each patient gave written consent to obtain blood and tissue samples that would otherwise have been discarded during surgery.

### 4.2. Quantitative Real-Time PCR

Total RNA was isolated from human right atrial samples using a commercially available kit. First-strand cDNA was synthesized from 1 mg of total RNA. cDNA was amplified using TaqMan master mix and primers from Thermo Fisher Scientific (Waltham, MA, USA) for the human glyceraldehyde-3-phosphate dehydrogenase (*GAPDH*): Hs00266705_g1; for human A_1_R (*ADORA1*): Hs00181231_m1; for human A_2A_R (*ADORA2A*): Hs00169123_m1; for human A_2A_B (*ADORA2B*): Hs00386497_m1; and for human A_3_R (*ADORA3*): Hs00181232_m1.

### 4.3. Patch-Clamp Technique

Isolated myocytes were subjected to the perforated patch technique using a HEKA EPC-10 amplifier (HEKA Elektronik, Lambrecht/Pfalz, Germany). Series resistance compensation was not applied. The extracellular solution contained (in mM): NaCl 127, TEA 5, HEPES 10, NaHCO_3_ 4, NaH_2_PO_4_ 0.33, glucose 10, pyruvic acid 5, CaCl_2_ 2, and MgCl_2_ 1.8 (pH = 7.4). The pipette solution contained (in mM): aspartic acid 109, CsCl 47, Mg_2_ATP 3, MgCl_2_ 1, na_2_phosphocreatine 5, Li_2_GTP 0.42, HEPES 10 (pH = 7.2 with CsOH), and 250 µg/mL amphotericin B. I_Ca_ density and properties were measured using a 200 ms depolarization from a holding potential of −80 mV to 0 mV. A 50 ms prepulse to −45 mV was used to inactivate the Na^+^ current. The I_Ca_ amplitude was normalized to the cell capacitance to obtain the I_Ca_ density. The decay of the I_Ca_ was fit with a double exponential to obtain the time constants tau-1 and tau-2 for fast and slow I_Ca_ inactivation. This study focused on the fast time constant, which is modulated by calcium release from the SR and by calcium entry through the L-type calcium channel. I_TI_ currents were recorded at a holding potential of −80 mV in 4 × 30 s intervals to determine the I_TI_ frequency. Brief exposure (6s) to 10 mM caffeine at a holding potential of −80 mV was used to release calcium from the SR and the time integral of the resulting transient inward NCX-current was used to assess the SR calcium load. Transformation of the charge carried by the NCX-current assumed a stoichiometry of 3 Na^+^:1 Ca^2+^ for the NCX. Working solutions containing 200 nM CGS21680 and/or 1 µM MRS1191 were prepared from 1 mM stock solutions dissolved in DMSO.

### 4.4. Immunofluorescent Labelling

Isolated myocytes were fixed and permeabilized, as previously described [32] and non-specific sites were blocked by incubation with PBS/Tween 20, 0.2% and horse serum, 10% for 30 min. Total and ser-2808 phosphorylated RyR2 were inmunofluorescently labeled with mouse anti-RyR2 (C3-33 NR07, 1:1200; Calbiochem, San Diego, CA, USA) and rabbit anti-ser2808-P (1:1200, A010-30, Badrilla, Leeds, UK). The secondary antibodies AlexaFluor 488 anti-mouse and AlexaFluor 594 anti-rabbit were diluted 1:1000 and used to stain total RyR2 green and ser-2808 phosphorylated RyR2 red. Images were acquired with a Leica AOBS SP5 confocal microscope (Wetzlar, Germany) and a 63× glycerol immersion objective.

### 4.5. Confocal Imaging

Confocal calcium images (512 × 140 pixels) were recorded at 90 Hz with the Leica SP5 AOBS resonance-scanning confocal microscope in fluo-4 loaded myocytes, as described previously [32]. Experiments were carried out at room temperature. Calcium sparks were detected and clustered in 2 × 2 µm^2^ regions of interest, termed spark sites, using a custom-made algorithm based on continuous wavelet transform of the temporal profile at every spatial location, as described elsewhere [33]. The calcium spark frequency and the number of spark sites were normalized to the cell area to obtain the calcium spark density (sparks/s/1000 µm^2^) and the spark site density (spark sites/1000 µm^2^). In addition, we calculated the number of sparks per site (sparks/site/s). A series of morphological features were measured for each spark signal: Relative amplitude of the peak to the local baseline (F/F0), full duration at half maximum (FDHM), decay constant of an exponential fit (tau), the coefficient of determination of the exponential fit (R2), and full width at half maximum (FWHM).

### 4.6. Data Analysis

Experimental data were collected and analyzed without knowledge about clinical data and clinicians did not know the experimental results. Statistical analysis was carried out using RStudio 4.2.2 statistical software. Unless otherwise stated, data were averaged for each patient and results are given as mean ± s.e.m. with indication of the number of patients in each group. Fisher’s exact test was carried out for categorical data. Student’s *t*-test was used for paired or unpaired comparisons, and ANOVA, ANOVA with Welch correction or Kruskal–Wallis were used for comparison of multiple effects, as indicated. Tests used are indicated for each figure and statistically significant effects are indicated with *p*-values or *: *p* < 0.05, **: *p* < 0.01; ***: *p* < 0.001.

## Figures and Tables

**Table 1:** Clinical characteristics of the study population.

		(53 Patients)
	Age, Years	67.0 [65.0; 69.0]
	Sex (Female/Male)	13/40 (24.5%/75.5%)
**Echocardiography**	LAD index	2.38 [2.26; 2.5]
	LVEF, %	55.0 [53.0; 57.0]
**Cardiovascular Risk Factors**	No smoking	22 (41.5%)
	Smoking	20 (37.7%)
	Ex smoking	11 (20.8%)
	Hypertension	36 (67.9%)
	Diabetes	13 (24.5%)
	Dyslipidemia	33 (62.3%)
**Surgical Treatment**	AVR	19 (35.8%)
	MVR	5 (9.4%)
	CABG	31 (58.5%)
**Pharmacological Treatment**	ACE inhibitor	22 (41.5%)
	Diuretics	20 (37,7%)
	ARB	13 (24.5%)
	Calcium antagonists	23 (43.4%)
	Nitrates	13 (24.5%)
	β-blockers	26 (49.0%)
	Acetylsalicylic acid	26 (49.0%)
	Statins	34 (64.1%)
	More than one treatment	49 (92.5%)

Categorical values are given as the number of patients with the condition and % of patients in parenthesis. Continuous values are given as mean ± standard error. Smoking was divided into three groups (non-, ex- and smokers). LAD, left atrial diameter; LAD index was obtained by normalizing LAD to the body mass index; LVEF, left ventricular ejection fraction; ACE inhibitor, angiotensin converting enzyme inhibitor; ARB, angiotensin receptor blocker; AVR, aortic valve replacement; MVR, mitral valve replacement; CABG, coronary artery bypass graft.

**Table 2:** Impact of the treatment with 200 nM CGS21680 (CGS) and 1 µM MRS1191 (MRS) on the incidence of calcium sparks and their properties in human atrial myocytes. Significant differences between treatment and control are indicated with * *p* < 0.05, ** *p* < 0.01, *** *p* < 0.001.

Frequencies	Control	CGS	CGS + MRS
Sparks/s/1000 µm^2^	0.017 ± 0.006	0.12 ± 0.04 ***	0.14 ± 0.06 **
Spark sites/1000 µm^2^	1.10 ± 0.42	6.18 ± 1.79 ***	3.51 ± 1.22 *
Sparks/site/s	0.018 ± 0.006	0.018 ± 0.003	0.031 ± 0.008
**Properties**			
Amplitude (∆F/F_0_)	1.06 ± 0.13	0.79 ± 0.06 *	0.69 ± 0.03 **
Tau (ms)	40.0 ± 6.6	35.2 ± 4.7	36.4 ± 4.3
FDHM (ms)	60.1 ± 8.3	60.2 ± 4.7	66.5 ± 5.2
FWHM (µm)	1.56 ± 0.18	1.49 ± 0.01	1.64 ± 0.16

## Data Availability

The data that support the findings of this study are available from the corresponding author upon reasonable request. Some data may not be made available because of privacy or ethical restrictions.
